# New virus of the family *Flaviviridae* detected in lumpfish (*Cyclopterus lumpus*)

**DOI:** 10.1007/s00705-017-3643-3

**Published:** 2017-11-17

**Authors:** Renate Hvidsten Skoge, Jarle Brattespe, Arnfinn Lodden Økland, Heidrun Plarre, Are Nylund

**Affiliations:** Department of Biology, University of Bergen, Thormøhlensgt. 55, Pb. 7803, 5020 Bergen, Norway

## Abstract

In this study, we determined the complete coding sequence of a putative new member of the family *Flaviviridae*, named “Cyclopterus lumpus virus” (CLuV), which is associated with a serious disease in lumpfish (*Cyclopterus lumpus*). The virus was present in all tissues tested, but pathology was primarily observed in the liver and kidneys. CLuV shows low but distinct similarity to the unassigned Tamana bat virus (TABV). Unlike other known members of the family *Flaviviridae*, translation of the entire CLuV polyprotein is dependent on a − 1 ribosomal frameshift in the NS2A region.

The copepod parasite *Lepeophtheirus salmonis* is a cause of major expense in the production of farmed Atlantic salmon (*Salmo salar*) in Norway [1, 2]. One widely used approach to control this parasite is the use of commercially farmed or wild cleaner fish, i.e., wrasse species and lumpfish (*Cyclopterus lumpus*) [3, 4]. A range of different pathogens have been detected in wild and farmed lumpfish, including viruses, bacteria and parasites [5–14]. In 2015, a new disease emerged in culture facilities for lumpfish, resulting in more than 50% mortality among young fish. Preliminary studies of these lumpfish showed the presence of some bacteria and parasites, but also a pathology that could not be attributed to any known pathogens. To gain more knowledge about this emerging disease, total RNA was isolated from the most strongly affected tissues (liver and kidney) of one lumpfish, and the RNA was used for Illumina sequencing. The resulting RNA sequences included the complete coding sequence of a putative new virus of the family *Flaviviridae*. The new member of the *Flaviviridae* presented in this study, “Cyclopterus lumpus virus” (CLuV), shows some similarity to Tamana bat virus (TABV) but only a distant relationship to members of existing genera. This study describes the genome of CLuV and the histopathology associated with the newly reported disease.

Lumpfish suffering from disease at a cultivation site in Western Norway were delivered live to the Fish Diseases Research Group at the Department of Biology, University of Bergen. A distinct finding during the dissection of the lumpfish was a pale and firm liver. Samples were taken from the gills, heart, liver, kidney and central nervous system (CNS) and stored in ethanol and in a modified Karnovsky fixative at 4 °C, or stored fresh at -85 °C. The fresh tissues were used for RNA extraction while the fixed tissues were processed and sectioned as described previously [15] and used for histology. Examination of the tissues from the moribund lumpfish revealed massive degeneration in the liver (Fig. 1A and B). The liver changes were associated with accumulation of large lipid droplets, which was also seen in the gills and kidney, but to a lesser extent (Fig. 1C and D).

**Fig. 1:**
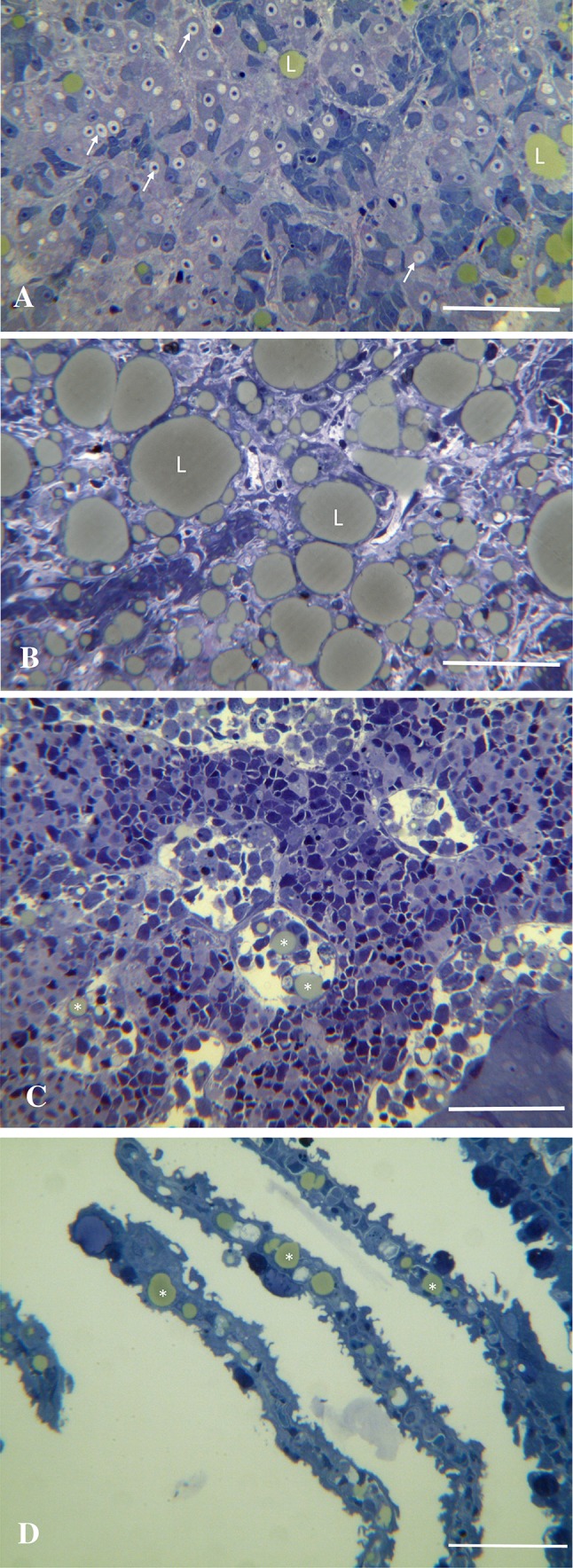
Liver and head kidney pathology associated with CLuV in lumpfish. **A**. Early stage of disease development showing degeneration of liver cells (arrows) and beginning accumulation of small lipid droplets (L). Bar = 50 μm. **B**. Terminal stage of the disease, showing massive changes in the liver and accumulation of large lipid (L) inclusions. Bar = 50 μm. **C**. Section through the head kidney of CLuV-infected lumpfish. Note the presence of cell-associated lipid droplets (asters) in the blood sinuses. Bar = 500 μm. **D**. Lipid droplets (asters) present in capillaries in gill lamellas. Bar = 500 μm

Attempts to cultivate the virus in CHSE-14 [16] and ASK [17] cells using sterile-filtered homogenates from the infected lumpfish as described previously [18] were unsuccessful. Therefore, infection studies using purified viruses could not be conducted and, hence, it could not be shown that CLuV was the cause of the described disease.

To determine the genome sequence of CLuV, liver and kidney samples from one lumpfish suffering from the putative new disease were homogenized in 1 ml of TRI Reagent® (Sigma-Aldrich) with a 5-mm bead for 7 min at 50 Hz in a TissueLyser LT (QIAGEN). Total RNA was extracted using a previously described protocol from [17], and one batch was kept at the laboratory for Sanger sequencing, while the other batch was sent to BaseClear for next-generation sequencing. Paired-end sequence reads (number of reads = 34,488,032) were generated using an Illumina HiSeq2500 system. FASTQ sequence files were generated using the Illumina Casava pipeline version 1.8.3. The quality of the FASTQ sequences was enhanced by trimming off low-quality bases using the “Trim sequences” option of the CLC Genomics Workbench version 9.5.1., and the quality-filtered sequence reads were assembled into 81,286 contig sequences using the “*de novo* assembly” option with a minimum allowed contig length of 120 base pairs. A BLASTN (version 2.6.0+) search was performed against the NCBI-NT database using a minimum e-value of 0.01, and the contigs were split into four files: a) vertebrates (48,545 contigs), b) viruses (2 contigs), c) others (529 contigs), and d) no BLAST hit (32,210 contigs). The sequences of the contigs were translated using ExPASy’s online translation tool (http://web.expasy.org/translate/), and the BLASTP algorithm of the BLAST suite was used to identify the putative amino acid sequences. One sequence (11,454 nt; accession no. MF776369) from the no-BLAST-hit group was identified as a possible new member of the virus family *Flaviviridae*. The complete coding sequence (10,451 nt) and parts of the non-coding 5’ and 3’ ends (242 and 467 nt, respectively) found by Illumina sequencing were confirmed by Sanger sequencing (in total, 11,160 nt).

RNA ligation was performed in an attempt to sequence the genome ends. Five μg of total RNA was incubated with 5 units of 5’ RNA pyrophosphohydrolase (NEB) in 40 μl of 1X NEBuffer 2 for 30 min at 37 °C [19], followed by RNA clean-up (RNeasy Mini Kit, QIAGEN). Four hundred ng of purified dephosphorylated RNA was ligated with 10 U of T4 RNA ligase (Thermo Scientific) in 20 μl of 1x reaction buffer with 0.1 mg of BSA per ml and 40 units of RNaseOUT™ (Invitrogen), for 1 h at 37 °C. Ligated RNA (2.5 μl) was used as template for reverse transcription (M-MLV RT, Promega) with primers annealing approximately 150-300 nt from the 3’end. The cDNA was then subjected to nested PCR (Expand™ High Fidelity PCR System, Roche), yielding products traversing the ligated genome ends. Finally, the PCR products were gel purified (QIAquick® Gel Extraction Kit, QIAGEN) and sequenced by the Sanger method. Some of the resulting sequences partially reflected the results from the Sanger sequencing of the non-ligated genome, while the majority gave a much shorter product containing only small parts of both the 5’ and 3’ non-translated regions (73 and 29 nt, respectively), giving a total sequence of only 10,553 nt. This discrepancy between the sequencing of ligated and non-ligated genomic RNA could be due to secondary structures in the genome ends interfering with RNA ligation, or it could indicate the existence of a hitherto unreported second population of truncated flavivirus RNA species in infected cells.

The genomes of members of the family *Flaviviridae* typically consists of a single positive-strand RNA of 9.5-12.5 knt that encodes a large polyprotein precursor that is cleaved into three structural (C, prM, and E) and seven non-structural (NS) proteins (NS1, NS2A, NS2B, NS3, NS4A, NS4B, and NS5) [20]. In the case of CLuV, the polyprotein is apparently encoded by two ORFs instead of the canonical single ORF. However, if the two ORFs were indeed translated separately, a large part of NS2A would not be translated. Programmed -1 ribosomal frameshifting is common for many members of the *Flaviviridae*, mostly to synthesize additional functional proteins, e.g., the overlapping *fifo* ORF in the NS2A/2B-coding region of insect-specific flavivirus genomes and the antigenic hepatitis C virus alternate reading frame protein (ARFP), but also to divert a proportion of the ribosomes for the synthesis of proteins encoded nearer the 5’ end [21–24]. For programmed -1 ribosomal frameshifting to occur, a “slippery” heptanucleotide consensus sequence and a downstream stimulatory RNA structure, separated by a spacer region (5-9 nt), must be present [23]. Indeed, just before the 3’ end of ORF1, there is a heptanucleotide sequence (_4034_ *G_GGU_UUU*_CCU_UAG_4047_-3’) fitting the consensus motif X_XXY_YYZ, where XXX can be any three identical nucleotides, YYY can be either AAA or UUU, Z can be either A, C or U, and underscores denote the reading frame. The consensus sequence is followed by 5 nt of spacing (_4041_CCUUA_4046_) and completed by a predicted stem-loop structure (_4046_GCUUCAUUCGCCUUUGAGCCCGCGACUGAAGC_4078_). Translation can then continue in the -1 frame, resulting in a complete flavivirus polyprotein.

Assuming that a ribosomal frameshift occurs, the best match for the 3483-amino-acid long CLuV polyprotein with Protein BLAST (NCBI) was unequivocally the TABV polyprotein, although the similarity was quite low (32% identity, 74% query cover). Given their similarities, the lengths of the putative CLuV proteins were estimated based on sequence alignment of CLuV with TABV (Vector NTI Suite 9.0.0). The putative capsid protein, C, was estimated to be 130 aa long. There were no significant hits for C in the GenBank database. The putative prM protein was estimated to be 208 aa long. This protein also did not match any proteins in the GenBank database, but a potential transmembrane region was detected (_177_IALLALVMAYFKVPTVHLLLIIL_199_). The prM in flaviviruses is cleaved by the host enzyme furin or an enzyme of similar specificity [25]. A possible cleavage site that conforms to the RXR/KR pattern [25] was identified (_131_RKTRNRR_137_), resulting in a putative pr protein of 71 aa containing one transmembrane region. The length of the putative envelope, E, protein of CLuV is 521 aa, and BLAST search gave a match of 24 and 22% with the E proteins from TABV and yellow fever virus, respectively. A SMART search (http://smart.embl-heidelberg.de) showed that this protein matches flavivirus glycoproteins and contains two putative transmembrane regions, _472_LFGMTSTIMMAVTGGAIVWLGTL_494_ and _501_NLTVIIGLILMAPLVITEV_519_ in the C-terminal end. A sequence of 14 aa, _108_DRGWTTGCFLFGQG_121_, that is possibly involved in fusion conforms to the DRGWXX(G/H)CXXFGKG motif observed in flaviviruses [26].

The putative NS1 protein is 329 aa long, and the sequence shows 37% identity to the NS1 from TABV. No domains, repeats, motifs or features could be predicted with confidence for NS1. Assuming that a ribosomal frameshift occurs, the continuous NS2A/NS2B polyprotein contains three putative transmembrane regions (401 aa long), one in NS2A (239-256) and two in NS2B (286-308 and 367-389), compared to five such regions in TABV. NS2A/NS2B from CLuV does not match any proteins in the GenBank database.

The putative 618-aa NS3 protein shows 39% sequence identity to TABV NS3. The NS3 protein of flaviviruses is hydrophilic, and the N-terminal part contains regions that have similarities to serine proteases, while the C-terminal domain contains regions with similarity to members of the DEAD family of RNA helicases. A region with strong similarity to the flavivirus DEAD domain (within a DEXDc domain, aa 1769-1932) is found in CLuV at position 1778-1918. The protein has a helicase C motif at position 339-460 and a putative single-stranded-nucleic-acid-binding domain (R3H) at position 319-383, as found in NS3 of other flaviviruses [27]. The flavivirus protease motif GXSGXP is also present in NS3 from CLuV as _128_GDSGSP_133_. Conserved motifs in NS3, based on alignment of CLuV with TABV are presented in Table 1. It was not possible to identify motifs IV and V in the helicase/NTPase by alignment of CLuV sequences with those of members of *Flaviviridae*. However, using a SMART search a sequence stretch containing motifs IV and V was identified as a helicase-C domain.

**Table 1:** Conserved motifs in the non-structural proteins NS3 and NS5 of CLuV

NS3				
Serine protease	Box 1	Box 2	Box 3	Box 4
TABV	GTLTTQYHVTCG	DYACYFGPW	GQSGTP	QEINGTLKPVALAGNSIVFG
CLuV	NIFHTMYHITKG	DWITYGGPW	GDSGSP	ARDADGTLTPVSLAGHTVPLQ
Helicase/NTPase	Motif I	Motif IA	Motif II	Motif III
TABV	VLKCGAGKTR	LVLVPTRVVANEAYNVLKD	NWQLIIVDESHFCNPETLALHN	MYLTATG
CLuV	VLRCGAGKTR	LLLAPTRTVAGEMYEALKD	NYKNIFVDEAHMQDPMTIALLG	FPMTATW
	Motif IV	Motif V	Motif VI	
TABV	IVYFVASGPEANEIAGKL	GLILTTNISEMGAN	SKIQRRGRVGR	
CLuV	RRSITFVPTIKKAEELYT	KPIAIFATNIAEVG	SKASTTQRRGR	
NS5				
Methyltransferase	Motif 1	Motif 2		
TABV	VVDGGCGAGGF	DTFVMDIGES		
CLuV	ILELGCGSGGF	DWVVMDIGEQ		
RNAd-RNApol	Motif A	Motif B	Motif C	Motif D
TABV	NWVIQDDTAGWDT	GTVVTYSMNTITN	SGDDCLLV	LKFINSTGFIRKDVPRH
CLuV	KAVIQDDTAAWDT	GTVVTYAMNTITN	SGDDMLFM	LKVINSLGFPRKGLTTW

Phobius and SMART searches identified eight putative transmembrane regions in the putative polyprotein NS4A-2K-NS4B (416 aa long). Three of these were located in the putative NS4A protein (128 aa long), one in 2K (25 aa long), and four in NS4B (263 aa long). A BLAST search using NS4A, 2K and NS4B, did not give any match with existing protein sequences. However, eight transmembrane regions were also predicted in this polyprotein in TABV. The NS5 protein of flaviviruses is a basic and hydrophilic protein that exhibits methyltransferase and RdRp activity. A protein matching NS5 is found at position 2624-3483 (859 aa). The conserved methyltransferase and RdRp motifs in NS5 are shown in Table 1.

To elucidate the evolutionary relationship between CLuV and known members of the *Flaviviridae*, reference sequences of *Flaviviridae* members were downloaded from the GenBank database, and Vector NTI Suite 9.0.0 was used to obtain multiple alignments of these sequences. To perform pairwise comparisons of the putative NS5 sequence from CLuV with those of other flavivirids, the multiple sequence alignment editor GeneDoc (www.psc.edu/biomed/genedoc) was used for manual adjustment of the sequence alignments. Ambiguously aligned regions were removed using Gblock [28]. This resulted in a sequence alignment of 751 amino acids (aa). Phylogenetic relationships were determined using the maximum-likelihood (ML) method available in TREE_PUZZLE 5.2 (http://www.tree-puzzle.de), employing the VT [29] model of amino acid substitution. The tree was constructed based on approximate maximum-likelihood values using the selected model of substitution and rate heterogeneity. The robustness of each node was determined using 10,000 replicates. A phylogenetic tree (Fig. 2) was drawn using TreeView [30] and showed that CLuV has the closest evolutionary relationship to TABV. TABV is considered as a potential member of the genus *Flavivirus*, but it is so distinct that it might be the founding member of a new genus [26]. Although CLuV is more closely related to TABV than to other members of the family *Flaviviridae*, the distance between CLuV and TABV is of such a proportion that CLuV could well be the first described virus of yet another genus.

**Fig. 2:**
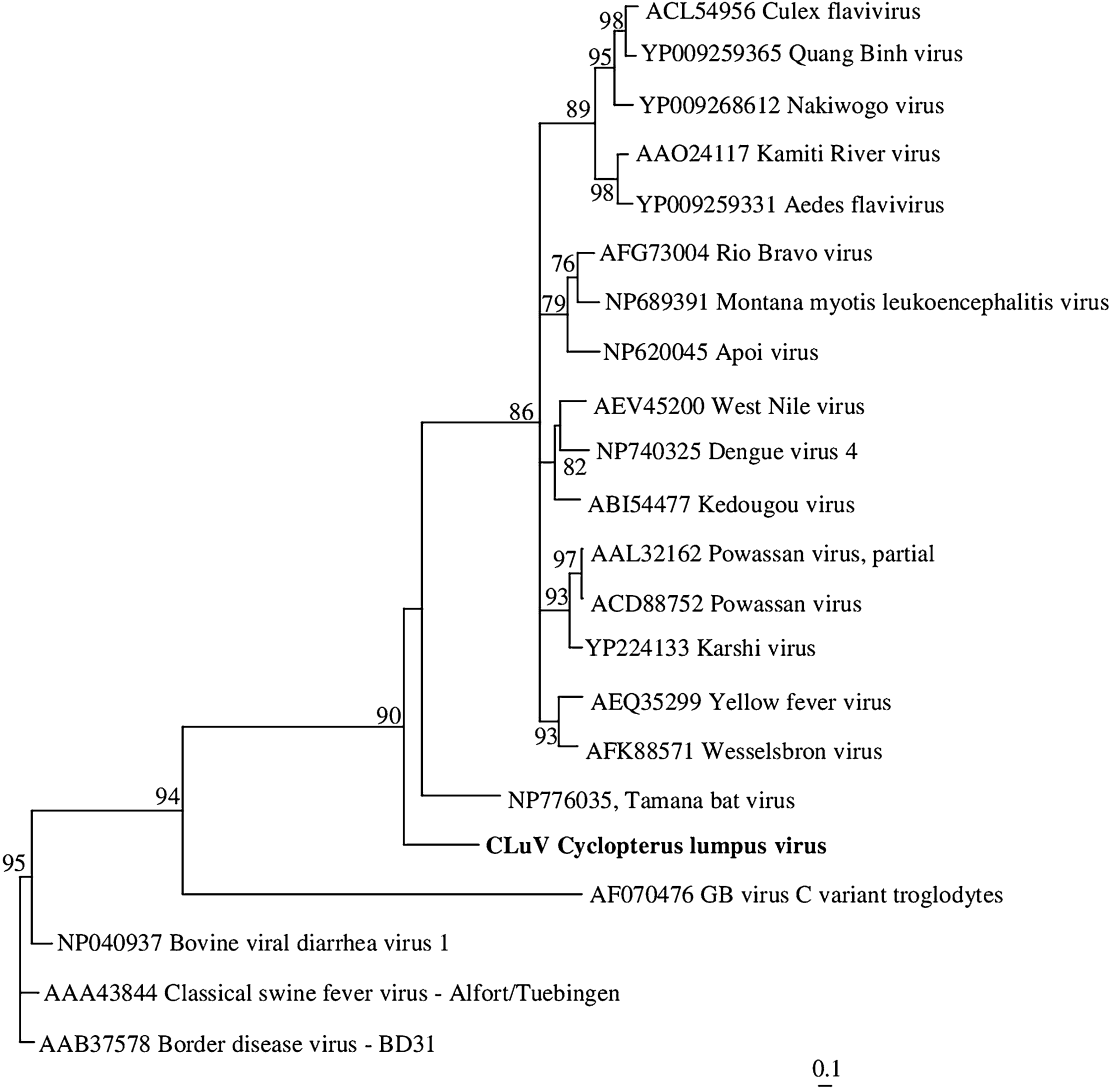
The phylogenetic position of CLuV (accession no. MF776369) in relation to selected members of the family *Flaviviridae* based on analysis of NS5 protein sequences (751 aa) after removal of ambiguously aligned regions using Gblocks [27]. The scale bar shows the number of amino acid substitutions as a proportion of the branch length

Real-time RT-PCR analyses using an assay (CLuV-EP) targeting the putative envelope protein gene (Cluv-ep-F: GCC GAG ACC TAT ATA ACT TGG AGA GA, Cluv-ep-Probe: ACC ACC CTC CAT TAC GTG A, Cluv-ep-R: CGA CGT TAT GGG CTT CTG AAA) showed the presence of the virus in all analyzed tissues from infected lumpfish (n = 37), with the highest amount detected in the liver (Ct value, 7.9). When wild and farmed lumpfish were screened using this assay, the virus was not detected in healthy fish (n = 22) or in fish where disease was caused by other pathogens (n = 34).

In conclusion, we have described the histopathology and genome of a flavivirus associated with a newly reported disease in farmed lumpfish. In CLuV, the polyprotein is encoded by two seemingly separate ORFs. However, unlike the additional ORFs reported for several other members of the *Flaviviridae*, translational slippage appears to be required for translation of the complete CLuV polyprotein, and especially the NS2A protein. The potential biological consequences of such a change in the expression pattern of a protein necessary for the production of mature viral particles remain to be investigated. The replacement of NS2A with a mutant containing a single amino acid substitution severely attenuated the production of viral particles of the flavivirus Kunjin virus [31]. Whether separate translation of the two CLuV ORFs would have a similar effect on the production of infectious virus particles requires further study. The presence of relatively large amounts of viral RNA in all tissues suggests that production of virions in these tissues is efficient. The fast spread of disease in lumpfish rearing tanks suggests that the virus might be spread by horizontal transmission and that CLuV, like TABV, could be considered a potential no-known-arthropod-vector (NKV) flavivirus [32]. Additional experiments are required to determine whether the virus is simply released from infected fish and transmitted orally to the next host or if transmission occurs via an arthropod vector. Furthermore, it needs to be ascertained that CLuV is indeed the causative agent of the reported disease in lumpfish. During the screening of wild and farmed lumpfish, CLuV was only detected in lumpfish suffering from the putative new disease and not in healthy lumpfish or in lumpfish suffering from other diseases. For future studies, detection of CLuV non-structural proteins by immunohistochemistry or their corresponding mRNAs by *in situ* hybridization could be used to investigate whether replication of the virus occurs in the affected tissues.
